# Acute hypoxia impairs uphill running performance and alters metabolic responses in adult trained trail runners: A pilot study

**DOI:** 10.14814/phy2.70996

**Published:** 2026-07-11

**Authors:** Diego Jaén‐Carrillo, Coralie Gobbo, Olivier Girard, Felipe García‐Pinillos

**Affiliations:** ^1^ Department of Sport Science University of Innsbruck Innsbruck Austria; ^2^ School of Human Sciences, Department of Sport Science, Exercise and Health University of Western Australia Perth Western Australia Australia; ^3^ Department of Physical Education and Sports, Faculty of Sport Sciences, Sport and Health University Research Institute (iMUDS) University of Granada Granada Spain; ^4^ Department of Physical Education, Sport and Recreation Universidad de La Frontera Temuco Chile

**Keywords:** blood lactate, female, oxygen uptake, simulated altitude, trail running, uphill running

## Abstract

To investigate the effects of acute hypoxia on uphill running and physiological responses during a 12‐min uphill time trial in trained trail runners and to assess potential sex differences. Thirteen trained trail runners (six females) completed a 12‐min treadmill uphill time trial (+12% incline) in normoxia and simulated hypoxia (~2500 m). Performance, physiological, and perceptual responses were recorded. Hypoxia reduced distance covered (−15%), speed (−15%), and power output (−8.5%) (all *p* < 0.001), while increasing respiratory exchange ratio and blood lactate concentration. Relative performance decrements were similar between sexes. Acute hypoxia impairs uphill running performance and increases metabolic strain irrespective of sex.

## Graphical abstract

Acute normobaric hypoxia (~2,500 m) reduced uphill 12‐min time‐trial performance (distance, speed, and power) by ~15% while increasing metabolic strain (higher RER and blood lactate, lower V̇O_₂_), with comparable responses in trained male and female trail runners.

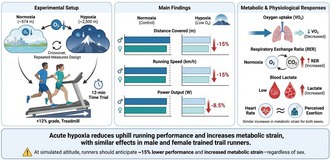

## 1 INTRODUCTION

Trail running involves foot races in natural environments (e.g., mountains, forests, or deserts), with minimal paved sections (~20%–25% of the course). A more extreme form, skyrunning, is performed at elevations ≥2000 m above sea level and features steep gradients often exceeding 30% (Scheer et al., 2020). Competition at moderate‐to‐high altitude (≥2500 m) imposes substantial physiological stress because the reduced partial pressure of oxygen lowers maximal work capacity and endurance performance (Gore et al., 1997), even in highly trained endurance athletes (Lawler et al., 1988).

These hypoxia‐related challenges may interact with sex‐specific physiological characteristics. Sex differences are more pronounced during uphill endurance exercise than on flat terrain, potentially due to differences in body composition, including a lower lean‐to‐fat mass ratio and a smaller proportion of fast‐twitch/type II fibers in females (Raberin et al., 2024). Additionally, ventilatory and oxygenation responses to hypoxia may differ between sexes, with females often experiencing greater exercise‐induced hypoxemia, likely due to diffusive limitations (Raberin et al., 2024). However, whether these mechanisms translate into meaningful performance differences during uphill running in hypoxia remains unclear.

Despite the growing popularity of trail running and skyrunning, the acute effects of hypoxia on uphill running performance and accompanying exercise responses, as well as the potential moderating role of sex, remain poorly understood in trained trail runners. Therefore, this study tested the hypothesis that acute normobaric hypoxia (simulated altitude of ~2500 m) would impair uphill running performance and increase cardio‐metabolic solicitation during a 12‐min uphill (+12%) time trial in trained trail runners, with comparable effects in males and females.

## 2 METHODS

### 2.1 Participants

Thirteen Tier‐3 trail runners (McKay et al., 2022) (age: 29 ± 5 years, VO_2_peak: 52.4 ± 6.3 mL·kg^−1^·min^−1^), including six females (Table 1), voluntarily participated in this study and provided written informed consent. A priori sample size calculation was performed using G*Power (v3.1) (Faul et al., 2007) for a paired *t*‐test, based on an expected large effect size (*d* = 0.85) consistent with previously reported reductions in endurance performance during acute hypoxia at similar altitudes (Gore et al., 1997; Mollard et al., 2007), with *α* = 0.05 and a target power of 80%, yielding a required sample of *n* = 13. Participants were recruited through convenience and snowball sampling via local trail running clubs and university sport networks. The study was approved by the ethics committee of the host university (No. 36/2023) and adhered to the principles of the Declaration of Helsinki.

**TABLE 1:** Descriptive characteristic of the participants (mean, SD).

	Males (*n* = 7)	Females (*n* = 6)
Height (cm)	179.1 (8.9)	165.0 (7.7)
Body mass (kg)	70.9 (6.7)	59.1 (4.5)
Heamoglobin (g/dL)	16.5 (0.6)	14.7 (0.6)
BMI (kg*m^−2^)	22.1 (1.3)	21.7 (0.8)
VO_2_peak (ml/min/kg)	56.4 (4.5)	47.6 (4.6)

Abbreviations: BMI, body max index; cm, centimeters; g/dL, grams per decilitre; kg, kilograms; VO_2_peak, peak oxygen consumption during the 12‐min time trial in normoxia.

### 2.2 Study design

A randomized, repeated‐measures design was used. Participants were fully familiarized with all testing procedures prior to experimentation and were instructed to refrain from intense physical activity for 48 h before each session. They attended two laboratory sessions, separated by 1 week, each involving a 12‐min uphill running time trial performed at a 12% gradient (hereafter referred to as the 12/12 running protocol, denoting 12 min of effort at 12% incline). In each trial, participants were instructed to cover the greatest possible distance under either normoxic (NOR; ~574 m) or hypoxic (HYP; simulated altitude ~2500 m) conditions.

### 2.3 Measurements

All trials were performed on an instrumented treadmill (HP Cosmos Pulsar 4P; HP Cosmos Sports and Medical, Germany) in an indoor laboratory maintained at 24°C. Before each trial, participants completed a standardized warm‐up consisting of 10 min of flat running at an easy long‐run pace.

Distance, speed, and mechanical power output were continuously monitored using a foot‐mounted power meter (Stryd power meter, Stryd NextGen, Boulder, CO, USA) clipped to the right shoe and paired with a tablet (iPad 10th gen., Apple Inc., Cupertino, CA, USA) (Taboga et al., 2022). Participants received time updates every 2 min.

Oxygen uptake and carbon dioxide production were measured using a respiratory gas analyzer (ML206, ADInstruments, Bella Vista, NSW, Australia) and a heated pneumotach system (Hans Rudolph Inc., Shawnee, KS, USA) connected to a gas mixing chamber (MLA246, ADInstruments). Respiratory exchange ratio (RER) was calculated as the ratio of carbon dioxide production to oxygen uptake (VCO_2_/VO_2_). Data were analyzed using LabChart Software (Version 8, ADInstruments). Before the hypoxia condition, participants stood for 5 min while breathing air corresponding to a simulated altitude of 2500 m to establish baseline values.

Heart rate was continuously recorded with a Polar belt monitor (Polar Electro, Kempele, Finland), and rating of perceived exertion (Borg 6–20 scale) was assessed immediately after each trial. Capillary blood samples (20‐μL) (E‐T‐E Capillaires, Hirschmann Laborgeräte GmbH, Eberstadt, Germany) were collected from the earlobe before and immediately after each trial, hemolyzed (Glucocapil reaction cup, Hitado GmbH, Möhnesee, Germany), and analyzed for blood lactate concentration using a calibrated analyzer (SUPER GL ambulance, Dr. Müller Gerätbau GmbH, Freital, Germany).

### 2.4 Statistical analysis

Data are presented as mean ± SD unless otherwise stated. Analyses were performed using GraphPad Prism (version 10.6.1; GraphPad Software, San Diego, CA, USA). Normality was assessed using the Shapiro–Wilk test, and homogeneity of variances with Levene's test. A two‐way ANOVA was conducted for each dependent variable, with condition (NOR vs. HYP) and sex (males vs. females) as fixed factors. When significant main effects or interactions were observed, Bonferroni‐adjusted post hoc comparisons were performed. Where appropriate, simple effects analyses examined differences within each sex. Statistical significance was set at *p* < 0.05.

## 3 RESULTS

**FIGURE 1:**
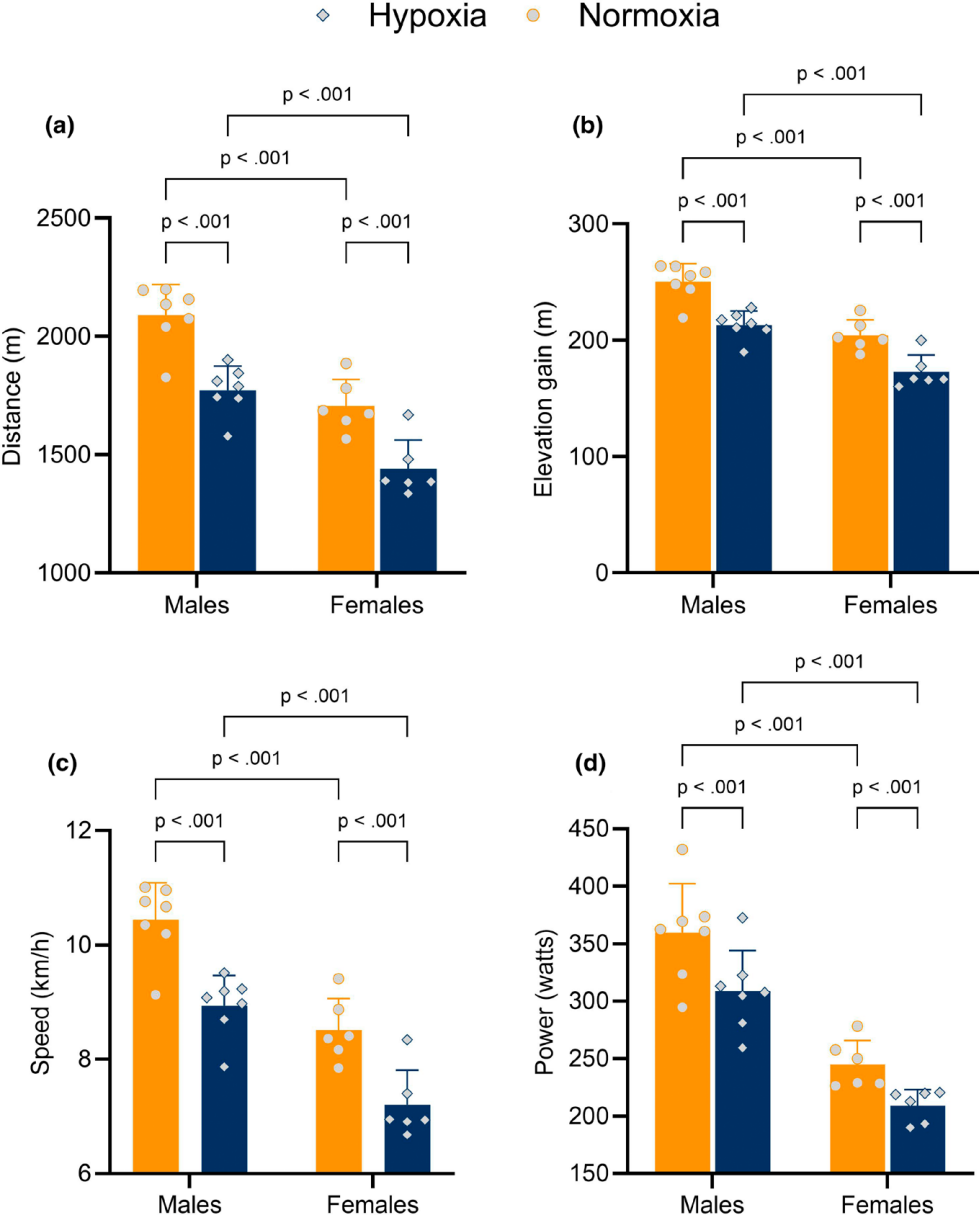
Uphill running performance outcomes (distance [a], elevation gain [b], speed [c], and power output [d]) in male and female trained trail runners under normoxic (NOR; ~574 m) and simulated hypoxic (HYP; ~2500 m) conditions. Bars represent mean ± SD with individual data points superimposed. Brackets indicate statistically significant post‐hoc differences with exact *p* values.

Running speed (8.1 ± 1.0 vs. 9.5 ± 1.1 km·h^−1^; −15%; *F* (1, 11) = 165.8, *p* < 0.001, ηp^2^ = 0.938) and power output (259 ± 67 vs. 283 ± 67 W; −8.5%; *F* (1, 11) = 165.0, *p* < 0.001, ηp^2^ = 0.938) were significantly lower in HYP than NOR (Figure 1). Similarly, distance covered (1606 ± 202 vs. 1898 ± 230 m; −15%; *F* (1, 11) = 151.3, *p* < 0.001, ηp^2^ = 0.932) and elevation gain (193 ± 31.7 vs. 227 ± 32.7 m; −15%; *F* (1, 11) = 150.4, *p* < 0.001, ηp^2^ = 0.932) were reduced in HYP.

**FIGURE 2:**
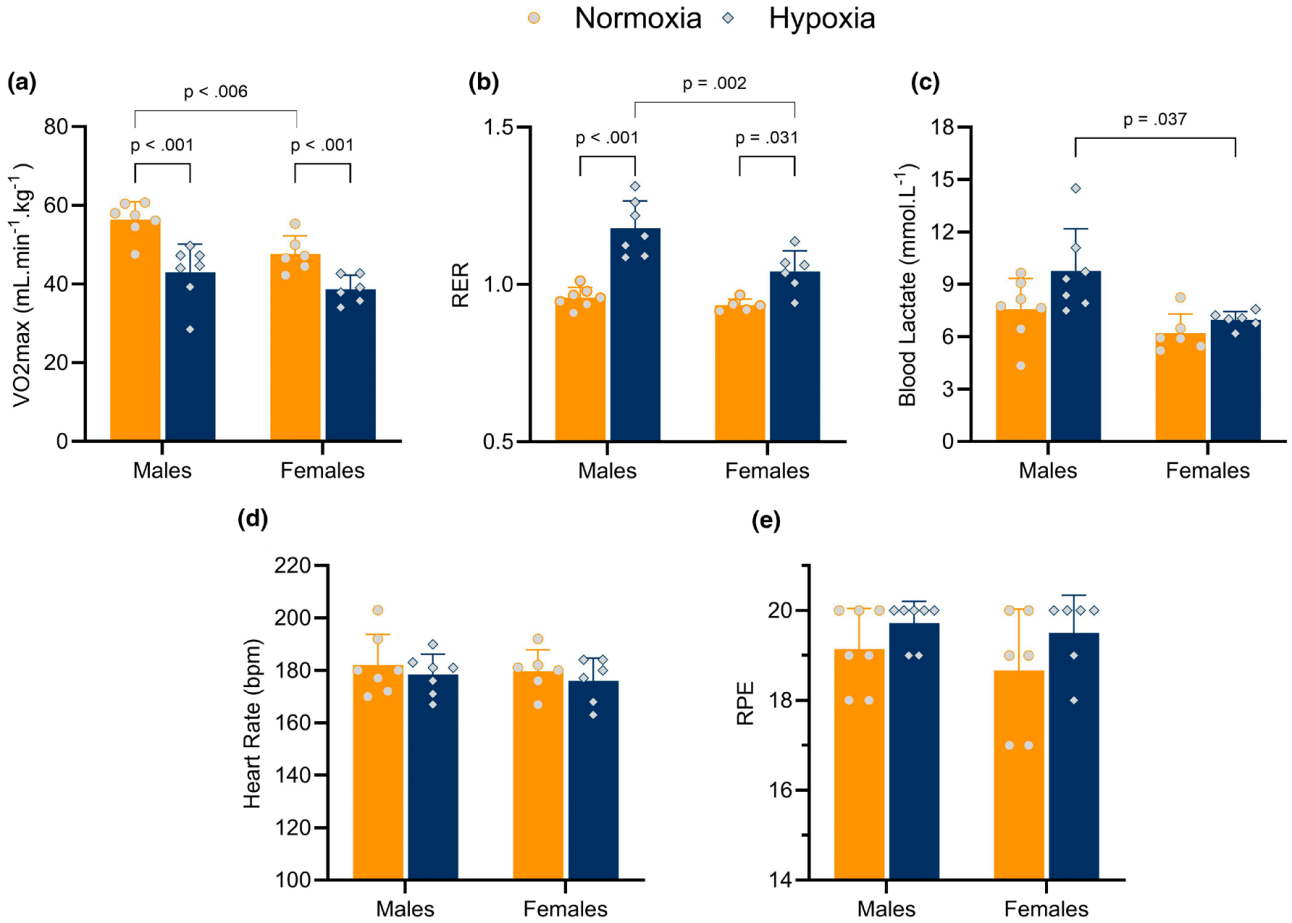
Metabolic (VO_2_ [a], respiratory exchange ratio [RER, b], blood lactate [c]), cardiovascular (heart rate [d]), and perceptual (ratings of perceived exertion [RPE, e]) responses to uphill running under normoxic and hypoxic conditions. Bars represent mean ± SD with individual data points superimposed. Brackets indicate statistically significant post‐hoc differences with exact *p* values.

Relative oxygen uptake was significantly lower in HYP compared with NOR (40.8 ± 4.5 vs. 52.0 ± 4.8 mL·kg^−1^·min^−1^; *F* (1, 11) = 137.8, *p* < 0.001, ηp^2^ = 0.926), whereas RER was higher (1.11 ± 0.10 vs. 0.95 ± 0.04; *F*(1, 21) = 45.5, *p* < 0.001, ηp^2^ = 0.79) (Figure 2). Blood lactate concentration was also elevated in HYP (8.4 ± 2.1 vs. 6.9 ± 1.7 mmol·L^−1^; *F* (1, 11) = 5.5, *p* = 0.039, ηp^2^ = 0.333).

Both trials were performed at near‐maximal intensity, as indicated by comparable rating of perceived exertion (19.6 ± 1 vs. 18.9 ± 1; *F* (1, 11) = 3.4, *p* = 0.092, ηp^2^ = 0.237) and maximal heart rate (177 ± 8 vs. 181 ± 10 bpm; *F* (1, 11) = 3.4, *p* = 0.090, ηp^2^ = 0.236) readings between HYP and NOR.

Under both NOR and HYP conditions, males showed significantly higher running speed, power output, distance covered, elevation gain, oxygen uptake, and blood lactate concentration than females (all *p* ≤ 0.037). However, the relative performance decrement induced by HYP was comparable between sexes, with both males and females showing similar reductions in running speed (−14% vs. −16%), power output (−14% vs. −14%), distance covered (−15% vs. −15%), and elevation gain (−14% vs. −16%), as well as oxygen uptake (−23% in both sexes). The only exception was RER, for which a significant condition × sex interaction was observed (*F*(1, 21) = 5.4, *p* = 0.030), with a greater hypoxia‐induced increase in males (post‐hoc Bonferroni: males NOR vs. HYP *p* < 0.001; females NOR vs. HYP *p* = 0.031).

## 4 DISCUSSION

Acute normobaric hypoxia (~2500 m) substantially impaired uphill running performance, with participants covering ~15% less distance at ~15% lower speed and ~ 8.5% lower power output compared to normoxia (Figure 1). Both trials elicited near‐maximal efforts, as indicated by comparable near‐ceiling ratings of perceived exertion and heart rate responses across conditions, indicating that the performance decrement reflected a genuine reduction in aerobic capacity rather than a conservative pacing strategy. These findings align with the ~10%–20% performance reductions reported during maximal aerobic exercise at similar simulated altitudes (~2500 m) (Calbet et al., 2003; Mollard et al., 2007). However, most previous studies have employed cycling ergometry or flat‐surface running, limiting direct comparison with uphill running.

A novel aspect of this study was the assessment of running power output during uphill running in hypoxia. Unlike speed alone, running power output integrates multiple biomechanical determinants and provides a more comprehensive measure of external load, particularly on inclines (García‐Pinillos et al., 2019; Taboga et al., 2022). Notably, the hypoxia‐induced decline in power output (~9%) was smaller than the reduction in speed (~15%), consistent with the non‐linear speed–power relationship in steep incline running. On steep gradients, a given change in metabolic demand translates into a disproportionately larger reduction in speed (Adejuwon et al., 2026; van Rassel et al., 2026), making power a more stable indicator of exercise intensity. The 12% gradient likely amplified the consequences of hypoxia, as uphill running substantially increases oxygen cost compared to flat running (van Rassel et al., 2026), leaving less physiological reserve available to compensate for the hypoxia‐induced reduction in O_2_ delivery (Calbet et al., 2003; Mollard et al., 2007).

The lower mean oxygen uptake observed under hypoxia (Figure 2) is consistent with the well‐established reduction in maximal oxygen uptake at altitude, primarily driven by reduced arterial O_2_ delivery and impaired aerobic capacity (Calbet et al., 2003; Mollard et al., 2007). Under self‐paced conditions, participants reduced speed to sustain near‐maximal effort, directly translating this reduced aerobic ceiling into the observed performance decrements. Males exhibited ~13% higher absolute oxygen uptake than females (Figure 2), consistent with evidence that sex differences in uphill endurance performance (18%–22%) exceed those observed on flat terrain (9%–12%) (Millet et al., 2024). The greater RER increase in males than females may reflect sex‐related differences in ventilatory response to hypoxia (Raberin et al., 2024); however, the absence of arterial blood gas measurements precludes definitive conclusions.

Several limitations should be acknowledged. First, the pilot‐level sample size (*n* = 13; seven males, six females) was calculated to detect main effects of condition and provided adequate power for this purpose (>99% for variables with ηp^2^ ≥0.79). However, statistical power to detect condition × sex interactions was limited: post‐hoc analysis indicated 23% power for medium‐sized interactions (ηp^2^ = 0.14) and 60% for large ones (ηp^2^ = 0.35), with 80% power only achievable for very large interactions (ηp^2^ ≥ 0.46). Accordingly, the null condition × sex findings for most variables should be interpreted cautiously, as they may partly reflect insufficient power rather than a true absence of sex‐specific responses. Second, the absence of arterial blood gas measurements precludes definitive conclusions regarding the mechanisms underlying the observed RER sex difference. Third, menstrual cycle phase and hormonal status were not controlled for in female participants, which may have introduced additional variability in physiological responses. Finally, the ecological validity of the protocol is constrained by the treadmill setting and fixed gradient, which may not fully replicate the variable terrain encountered in trail and sky running competitions. Future studies with larger, sex‐balanced samples are warranted to clarify potential sex‐specific adaptations to acute hypoxia during uphill running in ecological settings.

The relative hypoxia‐induced decrement was comparable between sexes (~14%–16% for speed, distance, and elevation gain; ~9% for running power output; ~23% for oxygen uptake). This indicates that male and female trail runners are equally susceptible to the performance‐impairing effects of normobaric hypoxia during uphill running. Accordingly, altitude‐based pacing adjustments should be adopted similarly across sexes.

## 5 PRACTICAL APPLICATIONS

- At altitude (~2500 m), expect a ~15% reduction in running speed and distance covered, and ~8.5% in power output, even during short maximal uphill efforts; adopt a more conservative pacing strategy accordingly.
- Prepare for greater metabolic strain at altitude, characterized by higher blood lactate concentration and increased reliance on anaerobic glycolysis, which may exaggerate fatigue and impair repeated efforts.
- Both male and female trail runners are equally susceptible to hypoxia‐induced performance decrements.

## 6 CONCLUSION

Acute normobaric hypoxia (~2500 m) substantially impairs uphill running performance and increases metabolic strain during a 12‐min maximal effort at 12% incline. These effects were comparable in magnitude between male and female trail runners, suggesting that altitude‐based pacing adjustments should be applied similarly regardless of sex.

## AUTHOR CONTRIBUTIONS

**Diego Jaén‐Carrillo:** Conceptualization; data curation; formal analysis; investigation; methodology; project administration; visualization. **Coralie Gobbo:** Investigation. **Olivier Girard:** Conceptualization; methodology; supervision. **Felipe García‐Pinillos:** Conceptualization; investigation; methodology; supervision.

## FUNDING INFORMATION

This research received no specific grant from any funding agency in the public, commercial, or not‐for‐profit sectors.

## CONFLICT OF INTEREST STATEMENT

The authors declare no conflict of interest.

## Data Availability

The data that support the findings of this study are available from the corresponding author upon reasonable request.

## References

[phy270996-bib-0001] Adejuwon, D. C. , Faricier, R. , & Keir, D. A. (2026). Running power at lactate threshold and lactate Turnpoint are unaltered by treadmill incline. International Journal of Sports Physiology and Performance, 1, 1–9. 10.1123/IJSPP.2025-0579 41921948

[phy270996-bib-0002] Calbet, J. A. L. , Boushel, R. , Rådegran, G. , Søndergaard, H. , Wagner, P. D. , & Saltin, B. (2003). Determinants of maximal oxygen uptake in severe acute hypoxia. American Journal of Physiology. Regulatory, Integrative and Comparative Physiology, 284(2), R291–R303. 10.1152/AJPREGU.00155.2002 12388461

[phy270996-bib-0003] Faul, F. , Erdfelder, E. , Lang, A. G. , & Buchner, A. (2007). G*power 3: A flexible statistical power analysis program for the social, behavioral, and biomedical sciences. Behavior Research Methods, 39(2), 175–191. 10.3758/BF03193146 17695343

[phy270996-bib-0004] García‐Pinillos, F. , Latorre‐Román, P. Á. , Roche‐Seruendo, L. E. , & García‐Ramos, A. (2019). Prediction of power output at different running velocities through the two‐point method with the Stryd™ power meter. Gait & Posture, 68, 238–243. 10.1016/J.GAITPOST.2018.11.037 30528962

[phy270996-bib-0005] Gore, C. J. , Little, S. C. , Hahn, A. G. , Scroop, G. C. , Norton, K. I. , Bourdon, P. C. , Woolford, S. M. , Buckley, J. D. , Stanef, T. , Campbell, D. P. , Watson, D. B. , & Emonson, D. L. (1997). Reduced performance of male and female athletes at 580 m altitude. European Journal of Applied Physiology and Occupational Physiology, 75(2), 136–143. 10.1007/S004210050138/METRICS 9118979

[phy270996-bib-0006] Lawler, J. , Powers, S. K. , & Thompson, D. (1988). Linear relationship between VO_2_max and VO_2_max decrement during exposure to acute hypoxia. Journal of Applied Physiology, 64(4), 1486–1492. 10.1152/JAPPL.1988.64.4.1486 3378983

[phy270996-bib-0007] McKay, A. K. A. , Stellingwerff, T. , Smith, E. S. , Martin, D. T. , Mujika, I. , Goosey‐Tolfrey, V. L. , Sheppard, J. , & Burke, L. M. (2022). Defining training and performance caliber: A participant classification framework. International Journal of Sports Physiology and Performance, 17(2), 317–331. 10.1123/ijspp.2021-0451 34965513

[phy270996-bib-0008] Millet, G. P. , Raberin, A. , Faiss, R. , Giovanelli, N. , Galindo, T. , Place, N. , & Sandbakk, Ø. (2024). Women upward‐sex differences in uphill performance in speed climbing, ski mountaineering, trail running, cross‐country skiing, and cycling. International Journal of Sports Physiology and Performance, 20(2), 246–255. 10.1123/IJSPP.2024-0354 39732139

[phy270996-bib-0009] Mollard, P. , Woorons, X. , Letournel, M. , Lamberto, C. , Favret, F. , Pichon, A. , Beaudry, M. , & Richalet, J. P. (2007). Determinants of maximal oxygen uptake in moderate acute hypoxia in endurance athletes. European Journal of Applied Physiology, 100(6), 663–673. 10.1007/S00421-007-0457-0/FIGURES/5 17534646

[phy270996-bib-0010] Raberin, A. , Burtscher, J. , Citherlet, T. , Manferdelli, G. , Krumm, B. , Bourdillon, N. , Antero, J. , Rasica, L. , Malatesta, D. , Brocherie, F. , Burtscher, M. , & Millet, G. P. (2024). Women at altitude: Sex‐related physiological responses to exercise in hypoxia. Sports Medicine, 54(2), 271–287. 10.1007/S40279-023-01954-6/FIGURES/3 37902936 PMC10933174

[phy270996-bib-0011] Scheer, V. , Basset, P. , Giovanelli, N. , Vernillo, G. , Millet, G. P. , & Costa, R. J. S. (2020). Defining off‐road running: A position Statement from the ultra sports science foundation. International Journal of Sports Medicine, 41(5), 275–284. 10.1055/A-1096-0980/ID/R7964-0019/BIB 32059243

[phy270996-bib-0012] Taboga, P. , Giovanelli, N. , Spinazzè, E. , Cuzzolin, F. , Fedele, G. , Zanuso, S. , & Lazzer, S. (2022). Running power: Lab based vs. portable devices measurements and its relationship with aerobic power. European Journal of Sport Science, 22(10), 1555–1568. 10.1080/17461391.2021.1966104 34420488

[phy270996-bib-0013] van Rassel, C. R. , Gow, S. , Watanabe, T. , Jaén‐Carrillo, D. , & MacInnis, M. J. (2026). Validity of Stryd running power for estimating metabolic demand during incline treadmill running. International Journal of Sports Physiology and Performance, 21(4), 597–603. 10.1123/IJSPP.2025-0382 41722548

